# Supplementary material for: Regulus infers signed regulatory relations from few samples’ information using discretization and likelihood constraints
Source: PLoS Comput Biol. 2024 Jan 22;20(1):e1011816. doi: 10.1371/journal.pcbi.1011816 (PMC10833539; doi:10.1371/journal.pcbi.1011816)
Supplement: S1 Fig — Top panel: the pool of entities is composed of 2 TFs, 2 regions and 2 genes. All of the biological entities activities or expression are first discretized (see Methods subsection Gene expression and region accessibility patterns). Middle panel: the interactions between the entities are then computed thanks to their inter-dependency and relations, using a Semantic Web framework. Region2 includes no binding site for TF2 therefore this interaction is discarded and Region1 is more than 500kb away from G2 and does not pass the distance threshold. Then, TF-region and region-gene relations are combined to obtain TF-region-gene relations resulting in a putative unsigned network. Bottom panel: relations are then filtered using the likelihood constraints. For each pair of TF expression and region accessibility patterns, the gene expression pattern is compared to the unique “perfect” gene expression pattern compatible either with an activation or an inhibition (see Methods subsection Regulatory likelihood constraints). To include some flexibility in the regulatory relations caused by modulation in TF expression or region accessibility, a relaxation parameter (δ) is introduced. Here the maximum allowed deviation (δ) from a perfect TF expression, gene expression and region accessibility patterns triple is 1. This step attributes a sign to the relation as an activation (+) or an inhibition (-). The result is a filtered and signed network (bottom right box). (PDF) [file pcbi.1011816.s001.pdf]

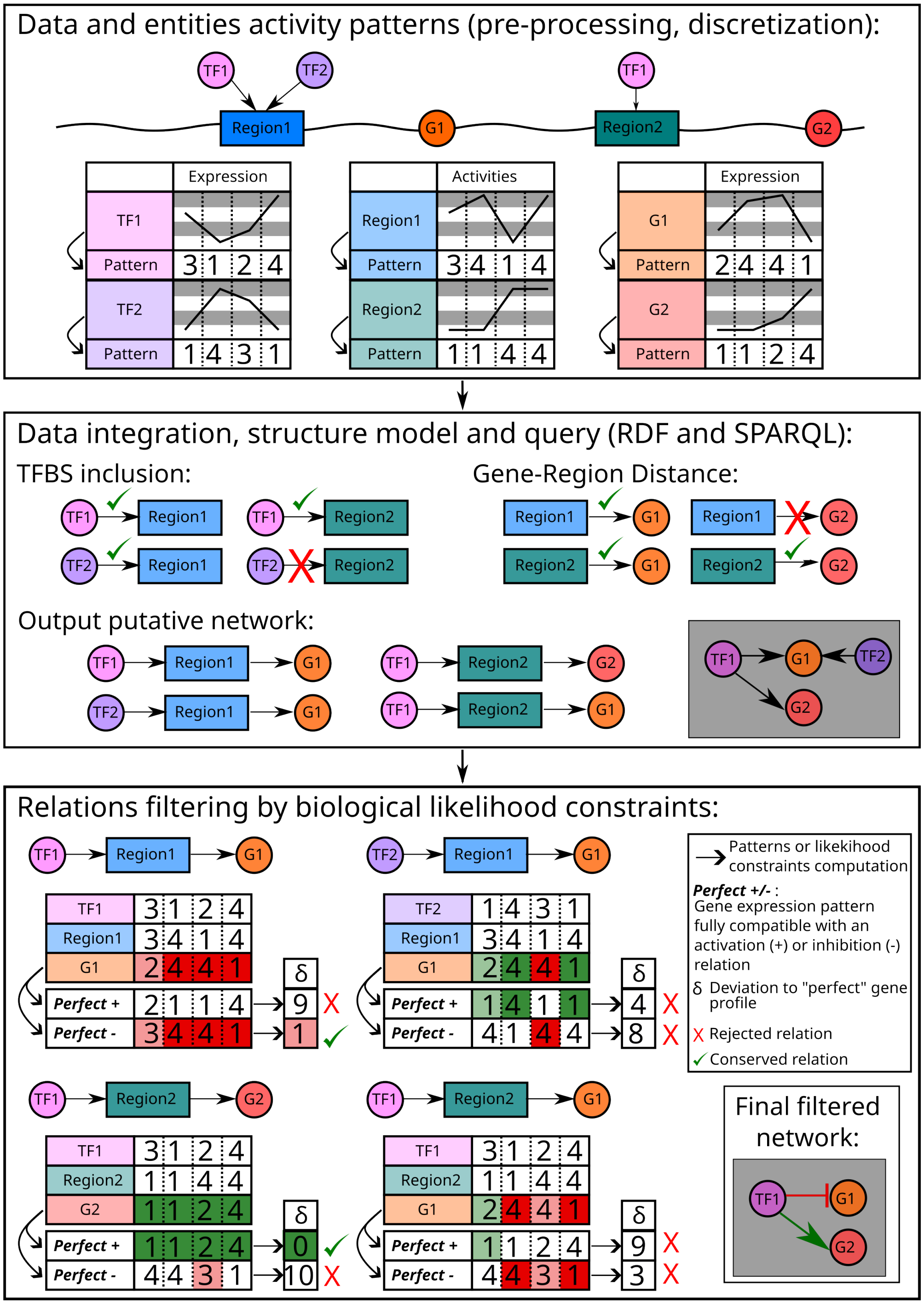

S1 Fig: **Toy-example of Regulus pipeline.** Top panel: the pool of entities is composed of 2 TFs, 2 regions and 2 genes. All of the biological entities activities or expression are first discretized (see Methods subsection *Gene expression and region accessibility patterns*).Middle panel: the interactions between the entities are then computed thanks to their inter-dependency and relations, using a Semantic Web framework. Region2 includes no binding site for TF2 therefore this interaction is discarded and Region1 is more than 500kb away from G2 and does not pass the distance threshold. Then, TF-region and region-gene relations are combined to obtain TF-region-gene relations resulting in a putative unsigned network. Bottom panel: relations are then filtered using the likelihood constraints. For each pair of TF expression and region accessibility patterns, the gene expression pattern is compared to the unique "perfect" gene expression pattern compatible either with an activation or an inhibition (see Methods subsection *Regulatory likelihood constraints*). To include some flexibility in the regulatory relations caused by modulation in TF expression or region accessibility, a relaxation parameter (δ) is introduced. Here the maximum allowed deviation (δ) from a perfect TF expression, gene expression and region accessibility patterns triple is 1. This step attributes a sign to the relation as an activation (+) or an inhibition (-). The result is a filtered and signed network (bottom right box).
