# Supplementary material for: Regulus infers signed regulatory relations from few samples’ information using discretization and likelihood constraints
Source: PLoS Comput Biol. 2024 Jan 22;20(1):e1011816. doi: 10.1371/journal.pcbi.1011816 (PMC10833539; doi:10.1371/journal.pcbi.1011816)
Supplement: S2 Fig — (a-b) For each variable and significant (over the minimal expression / activity threshold) gene expression or regulatory region activity of our B cell dataset, the number of equal bins necessary to separate all values between the minimum and the maximum was computed. The absolute frequency of this bins number is reported for genes (a) and regions (b). For most genes and regions, their activity over the samples is described by using three to five bins, with a peak at four bins. Relative to Figs 1 and 5 and Methods subsection Gene expression and region accessibility patterns. (PDF) [file pcbi.1011816.s002.pdf]

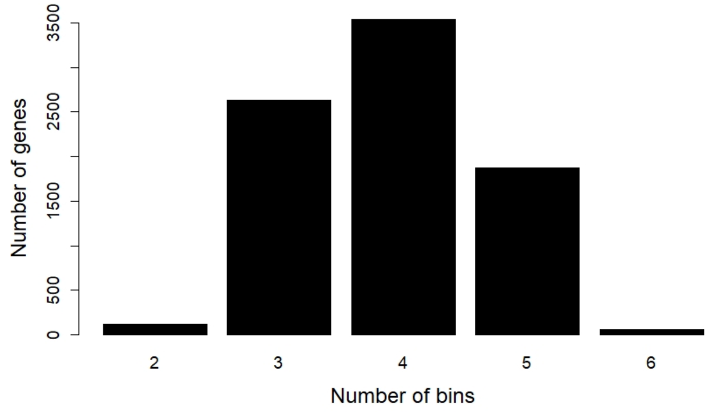

(a)

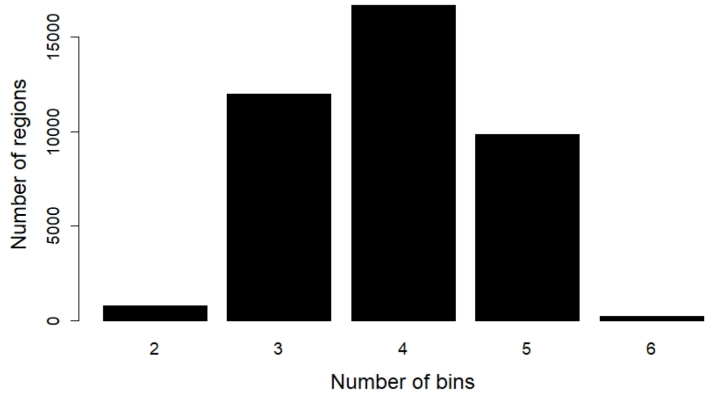

(b)

S2 Fig: **Number of bins for computing the activity patterns.** (a-b) For each variable and significant (over the minimal expression / activity threshold) gene expression or regulatory region activity of our B cell dataset, the number of equal bins necessary to separate all values between the minimum and the maximum was computed. The absolute frequency of this bins number is reported for genes (a) and regions (b). For most genes and regions, their activity over the samples is described by using three to five bins, with a peak at four bins. Related to Figs 1 and 5 and Methods subsection *Gene expression and region accessibility patterns.*
