# Supplementary material for: Regulus infers signed regulatory relations from few samples’ information using discretization and likelihood constraints
Source: PLoS Comput Biol. 2024 Jan 22;20(1):e1011816. doi: 10.1371/journal.pcbi.1011816 (PMC10833539; doi:10.1371/journal.pcbi.1011816)
Supplement: S3 Fig — Example of the data structure obtained after applying Semantic Web technologies integration to B cells genomic datasets. Relative to Fig 1, Results subsection The Regulus tool and Methods subsection Data graph for integration and query. (PDF) [file pcbi.1011816.s003.pdf]

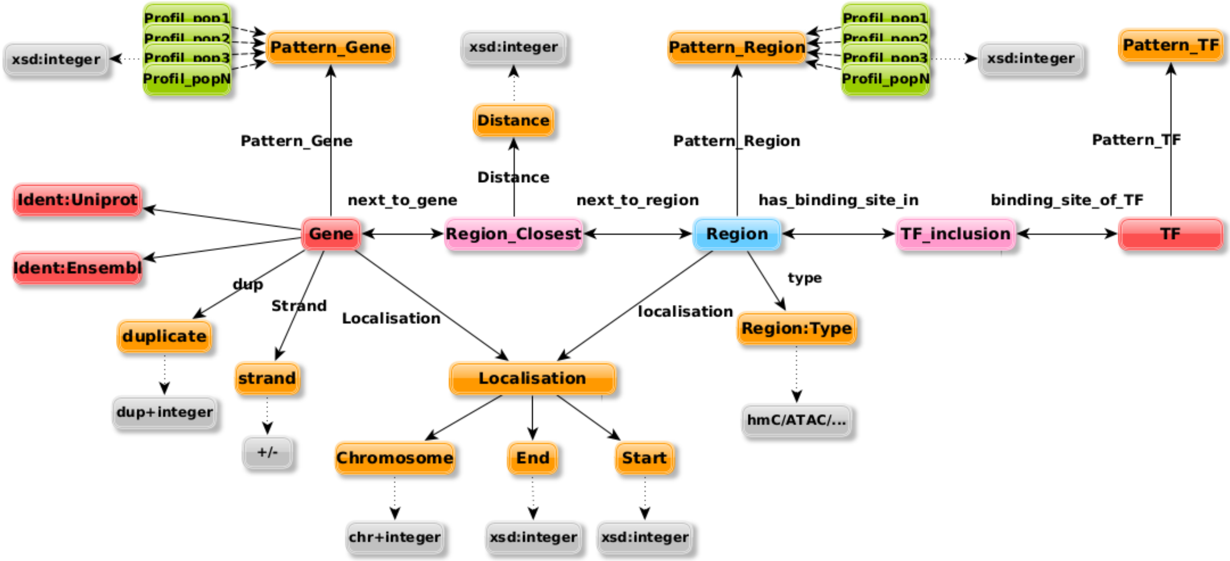

S3 Fig: **RDF data structure model after integration of all pre-processed input data.** Example of the data structure obtained after applying Semantic Web technologies integration to B cells genomic datasets. Relative to Fig 1, Results subsection *The Regulus tool* and Methods subsection *Data graph for integration and query*.
