# Supplementary material for: Regulus infers signed regulatory relations from few samples’ information using discretization and likelihood constraints
Source: PLoS Comput Biol. 2024 Jan 22;20(1):e1011816. doi: 10.1371/journal.pcbi.1011816 (PMC10833539; doi:10.1371/journal.pcbi.1011816)
Supplement: S4 Fig — Relative to Fig 1, Results subsection The Regulus tool and Methods subsection Data graph for integration and query. (PDF) [file pcbi.1011816.s004.pdf]

```
PREFIX : <http://www.semanticweb.org/user/ontologies/2018/1#>
PREFIX rdf: <http://www.w3.org/1999/02/22-rdf-syntax-ns#>
PREFIX rdfs: <http://www.w3.org/2000/01/rdf-schema#>

SELECT DISTINCT ?Gene ?Pattern_Gene ?Region
    ?Pattern_Region ?TF ?Pattern_TF
WHERE {
    ?Gene_uri :next_to_gene ?Region_Closest_uri .
    ?Region_Closest_uri :next_to_region ?Region_uri .
    ?TF_inclusion_uri :has_binding_site_in ?Region_uri .
    ?TF_inclusion_uri :binding_site_of_TF ?TF_uri .
    ?Gene_uri rdf:type :Gene .
    ?Gene_uri rdfs:label ?Gene .
    ?Gene_uri :PatternGene ?Pattern_GeneCategory .
    ?Pattern_GeneCategory rdfs:label ?Pattern_Gene .
    ?Region_Closest_uri rdf:type :Region_Closest .
    ?Region_Closest_uri rdfs:label ?Region_Closest .
    ?Region_Closest_uri :Distance ?Region_Closest_Distance .
    ?Region_uri rdf:type :Region .
    ?Region_uri rdfs:label ?Region .
    ?Region_uri :Pattern_Region ?Pattern_RegionCategory .
    ?Pattern_RegionCategory rdfs:label ?Pattern_Region .
    ?TF_inclusion_uri rdf:type :TF_inclusion_ATAC .
    ?TF_inclusion_uri rdfs:label ?TF_inclusion .
    ?TF_uri rdf:type :Transcription_Factor .
    ?TF_uri rdfs:label ?TF .
    ?TF_uri :PatternTF ?Pattern_TFCategory .
    ?Pattern_TFCategory rdfs:label ?Pattern_TF .
    FILTER ( ?Region_Closest_Distance < 500000 ) .

}
```

S4 Fig: **SPARQL query** used to retrieve all relations between TF, Region and Gene entities and their associated activity patterns. Relative to Fig 1, Results subsection *The Regulus tool* and Methods subsection *Data graph for integration and query.*
