# Supplementary material for: Regulus infers signed regulatory relations from few samples’ information using discretization and likelihood constraints
Source: PLoS Comput Biol. 2024 Jan 22;20(1):e1011816. doi: 10.1371/journal.pcbi.1011816 (PMC10833539; doi:10.1371/journal.pcbi.1011816)
Supplement: S5 Fig — Biological likelihood constraints threshold tables divided by relation qualification as activation (left column) or inhibition (right column) and by region pattern value from 4 (highest accessibility value) to 1 (bottom, less accessible). Without deviation (δ = 0), a relation is accepted only if for all cell populations, the TF expression, gene expression and region accessibility patterns triple satisfy Eq 1 in Methods subsection Regulatory likelihood constraints, corresponding to the dark blue cells. With a deviation of one (δ = 1), relations are accepted if at most one cell population satisfy Eqs 2 and 3 (light blue cells), and all other cell populations satisfy Eq 1. A deviation of 1 and the relaxed constrain on the region (δ = 1_regOFF) allows for only one cell population satisfying Eq 2 but not Eq 3 (light blue or gray cells) and the rest satisfying Eq 1. A deviation of two (δ = 2) allows for either one cell population satisfying none of the Equations above (white cells) or two cell populations satisfying both Eqs 2 and 3 (light blue cells). Relative to Fig 1 and Methods subsection Regulatory likelihood constraints. (PDF) [file pcbi.1011816.s005.pdf]

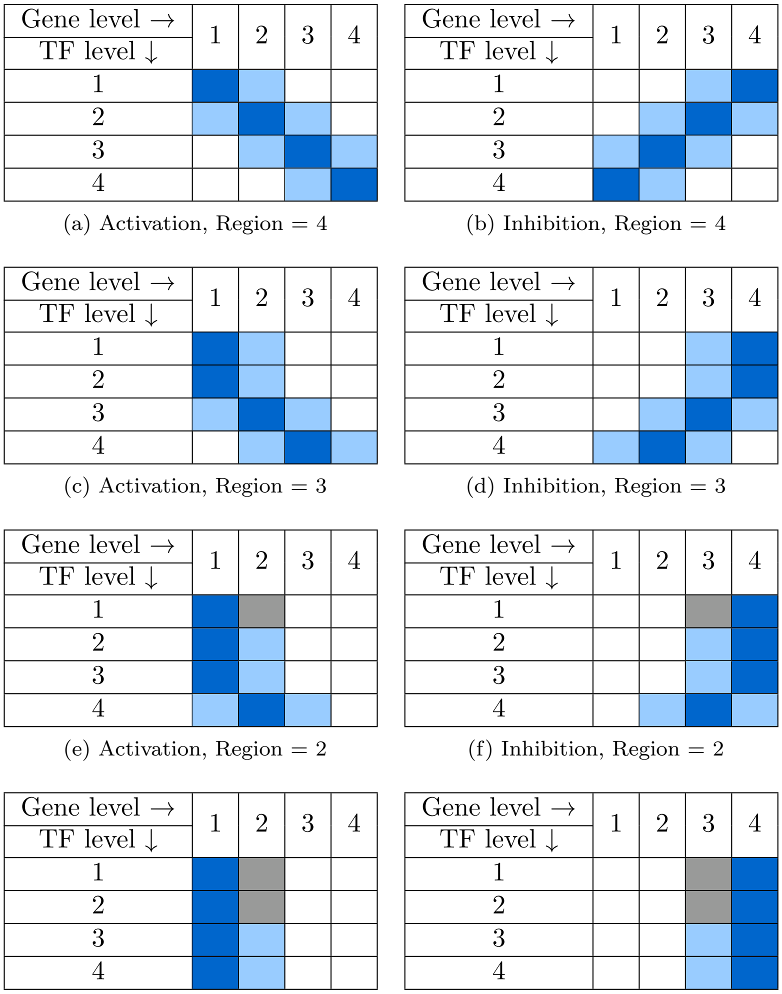

(a) Activation, Region = 4 (b) Inhibition, Region = 4

(c) Activation, Region = 3 (d) Inhibition, Region = 3

(e) Activation, Region = 2 (f) Inhibition, Region = 2

(g) Activation, Region = 1 (h) Inhibition, Region = 1

S5 Fig: **Biological likelihood constraints tables** divided by relation qualification as activation (left column) or inhibition (right column) and by region pattern value from (highest accessibility value) to 1 (bottom, less accessible). Without deviation (δ=0), a relation is accepted only if for all cell populations, the TF expression, gene expression and region accessibility patterns triple satisfy Eq 1 in Methods subsection *Regulatory likelihood constraints*, corresponding to the dark blue cells. With a deviation of one (δ=1), relations are accepted if at most one cell population satisfy Eqs 2 and 3 (light blue cells), and all other cell populations satisfy Eq 1. A deviation of 1 and the relaxed constrain on the region ($\delta = 1$_regOFF) allows for only one cell population satisfying Eq 2 but not Eq 3 (light blue or gray cells) and the rest satisfying Eq 1. A deviation of two (δ=2) allows for either one cell population satisfying none of the Equations above (white cells) or two cell populations satisfying both Eqs 2 and 3 (light blue cells). Related to Fig 1 and Methods subsection *Regulatory likelihood constraints*.
