# Supplementary material for: Regulus infers signed regulatory relations from few samples’ information using discretization and likelihood constraints
Source: PLoS Comput Biol. 2024 Jan 22;20(1):e1011816. doi: 10.1371/journal.pcbi.1011816 (PMC10833539; doi:10.1371/journal.pcbi.1011816)
Supplement: S6 Fig — Constitution of the Regulus networks while shifting the deviation (δ) level: 0 is the more stringent and 2 the more lenient one, deviation 1 and relaxed region constraints (δ = 1_regOFF) represent an offset of 1 all over the diagonal in the biological likelihood constraints table (see S5 Fig). The choice of a deviation of two lead to regulatory networks 2.4 to 7.4 times bigger than the most stringent one. We can also note that more relaxed constraints favor inhibition (“-”) relations. Relative to Results subsection Application to FANTOM5 data. (PDF) [file pcbi.1011816.s006.pdf]

Sub-set	Deviation Region cstr ON	Nb relations TF-region-Gene Predicted before filtering	Nb relations TF-region-Gene After filtering	Nb relations TF-Gene After filtering	Ratio relations After filtering Over δ=0	Nb relations TF-Gene "+"	Nb relations TF-Gene "-"	Ratio relations TF-Gene +/-
**Dataset 1**	δ=0	3,005,934	120,072	90,640	1	67,908	22,732	3
	δ=1		219,495	164,251	1.81	114,624	49,627	2.3
	δ=1_regOFF		230,335	171,401	1.89	119,313	52,088	2.3
	δ=2		492,961	352,752	3.89	237,529	115,224	2.1
**Dataset 2**	δ=0	3,005,934	183,889	135,156	1	128,558	6,598	19.5
	δ=1		237,487	178,514	1.32	154,276	24,238	6.3
	δ=1_regOFF		242,176	181,966	1.35	156,366	25,600	6.1
	δ=2		446,271	323,390	2.39	244,810	78,576	3.1
**Dataset 3**	δ=0	3,005,934	69,587	53,105	1	37,660	15,445	2.4
	δ=1		165,804	125,451	2.36	79,359	46,092	1.7
	δ=1_regOFF		177,376	133,614	2.52	84,268	49,346	1.7
	δ=2		510,855	376,856	7.10	224,112	152,744	1.5
**Dataset 4**	δ=0	3,005,934	71,493	55,041	1	42,215	12,826	3.3
	δ=1		165,597	126,145	2.29	88,609	37,536	2.3
	δ=1_regOFF		182,740	138208	2.51	95,464	42,744	2.2
	δ=2		545,080	406,616	7.39	260,599	146,017	1.8

(a) Number of relations by network after *Regulus*, on the 4 sub-sets of *Fantom5*.

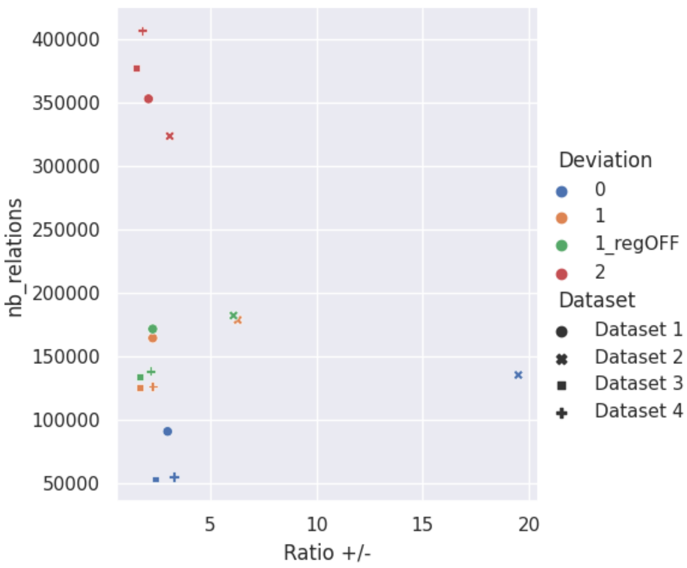

(b) Relation number over ration of activation ("+")/inhibition ("-") relations. Based on Table **S6 Fig**a

S6 Fig: **Construction of regulatory networks while varying the likelihood constraints deviation.** Constitution of the *Regulus* networks while shifting the deviation (δ) level: 0 is the more stringent and 2 the more lenient one, deviation 1 and relaxed region constraints (δ = 1_regOFF) represent an offset of 1 all over the diagonal in the biological likelihood constraints table (see S5 Fig). The choice of a deviation of two lead to regulatory networks 2.4 to 7.4 times bigger than the most stringent one. We can also note that more relaxed constraints favor inhibition ("-") relations. Relative to Results subsection *Application to FANTOM5 data*.
