# Supplementary material for: Regulus infers signed regulatory relations from few samples’ information using discretization and likelihood constraints
Source: PLoS Comput Biol. 2024 Jan 22;20(1):e1011816. doi: 10.1371/journal.pcbi.1011816 (PMC10833539; doi:10.1371/journal.pcbi.1011816)
Supplement: S7 Fig — Percentage of genes from Roadmap Epigenomics RNA-seq datasets related to the cell populations found in circuits inferred by Regulus using four different constraints deviation settings (same as S6 Fig), genes have been separated by their level of expression as in Figs 2F, 3C and 3D. The lowly expressed genes are slightly (but not significantly) better recovered when including a deviation (irrespective of its value), while middle and high expressed genes better recovered. Relative to Results subsection Application to FANTOM5 data. (PDF) [file pcbi.1011816.s007.pdf]

Regulated in	Deviation	Top 10%	Mid 10%	Bottom 10%
**Dataset 1**	δ=0	81	75	36
	δ=1	90.75	86.25	41
	δ=1_regOFF	91.75	86.5	41.25
	δ=2	94	92	45
**Dataset 2**	8	74.75	71.75	25.25
	δ=1	90	88.25	32.5
	δ=1_regOFF	90.75	88.5	32.5
	δ=2	94.5	93	34.25
**Dataset 3**	8	68	67	43
	δ=1	89	88	49.25
	7_reg1	89.75	88.5	49.5
	δ=2	93	92	51
**Dataset 4**	8	65.25	64.25	47.5
	δ=1	88.75	85.75	55
	δ=1_regOFF	89.5	87	55.25
	δ=2	93.25	91	56.5

(a) Percentage of genes from the RNA-seq related to the tissues found in the resulting networks. The RNA-seq genes are separated in three categories: the top 10% most expressed, the middle 10% and the 10%least expressed.

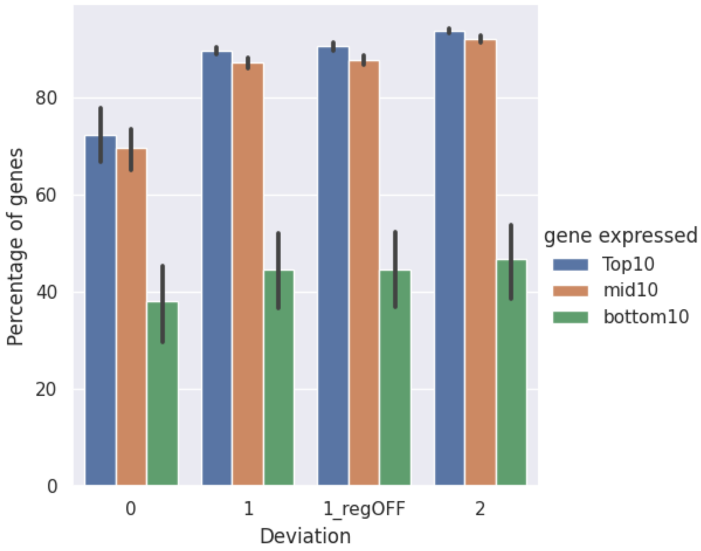

(b) Percentage of genes from the RNA-seq related to the tissues found in the resulting networks. Based on Table **S7 Fig**a

S7 Fig: **Effect of likelihood constraints deviation on the recovery of known expressed gene.** Percentage of genes from *Roadmap Epigenomics* RNA-seq datasets related to the cell populations found in circuits inferred by *Regulus* using four different constraints deviation settings (same as S6 Fig), genes have been separated by their level of expression as in Figs 2F, 3C and 3D. The lowly expressed genes are slightly (but not significantly) better recovered when including a deviation (irrespective of its value), while middle and high expressed genes better recovered. Relative to Results subsection *Application to FANTOM5 data.*
