# Supplementary material for: Regulus infers signed regulatory relations from few samples’ information using discretization and likelihood constraints
Source: PLoS Comput Biol. 2024 Jan 22;20(1):e1011816. doi: 10.1371/journal.pcbi.1011816 (PMC10833539; doi:10.1371/journal.pcbi.1011816)
Supplement: S8 Fig — For each deviation (same as S6 Fig): number of relations which present in the different relations databases (Cytreg, HTRI, Signor, Tfacts or Trrust) or at least in one (ALL). The proportion of the networks recovered is between 0.4 and 0.85% with an average of 0.56%, this number slightly lowers with a relaxed deviation but with no significance (0.6 at δ = 0 and 0.51% at 2). Relative to Results subsection Application to FANTOM5 data. (PDF) [file pcbi.1011816.s008.pdf]

| Dataset | Deviation | Cytreg | HTRI | Signor | Tfacts | Trrust | ALL |
|---|---|---|---|---|---|---|---|
| **Dataset 1** | Reachable | 920 | 12985 | 867 | 2339 | 4216 | 18925 |
| | $\delta$=0 | 47 | 195 | 43 | 132 | 217 | 511 |
| | P-Value | 8.59e-22 | 4.60e-14 | 1.29e-19 | 7.33e-63 | 8.96e-95 | 7.80e-110 |
| | $\delta$=1 | 62 | 232 | 86 | 219 | 732 | 936 |
| | P-Value | 2.89e-21 | 7.19e-03 | 1.29e-40 | 6.71e-96 | 0 | 2.01e-202 |
| | $\delta$=1_regOFF | 103 | 362 | 93 | 262 | 454 | 1001 |
| | P-Value | 2.76e-51 | 2.64e-23 | 5.30e-45 | 1.48e-127 | 6.53e-213 | 8.44e-224 |
| | $\delta$=2 | 176 | 836 | 179 | 457 | 867 | 1964 |
| | P-Value | 2.98e-74 | 7.14e-71 | 1.89e-80 | 4.34e-193 | 0 | 0 |
| **Dataset 2** | Reachable | 937 | 13995 | 1068 | 2689 | 4938 | 20866 |
| | $\delta$=0 | 10 | 183 | 50 | 159 | 305 | 540 |
| | P-Value | **NS** | **NS** | 1.13e-14 | 3.09e-55 | 8.70e-109 | 2.82e-52 |
| | $\delta$=1 | 14 | 176 | 74 | 197 | 408 | 852 |
| | P-Value | **NS** | **NS** | 1.14e-23 | 1.10e-63 | 1.26e-146 | 2.81e-118 |
| | $\delta$=1_regOFF | 26 | 381 | 75 | 228 | 415 | 884 |
| | P-Value | 1.06e-02 | 1.04e-18 | 8.00e-24 | 2.41e-83 | 8.50e-149 | 9.58e-127 |
| | $\delta$=2 | 83 | 914 | 143 | 390 | 730 | 1805 |
| | P-Value | 2.81e-17 | 2.82e-98 | 3.63e-47 | 6.27e-136 | 1.77e-257 | 0 |
| **Dataset 3** | Reachable | 811 | 4884 | 940 | 2500 | 4473 | 11197 |
| | $\delta$=0 | 38 | 100 | 42 | 102 | 158 | 317 |
| | P-Value | 2.18e-24 | 3.65e-31 | 5.29e-26 | 4.29e-57 | 8.55e-79 | 1.97e-130 |
| | $\delta$=1 | 61 | 205 | 81 | 182 | 369 | 744 |
| | P-Value | 3.68e-29 | 1.63e-52 | 2.98e-42 | 3.19e-81 | 9.77e-180 | 1.77e-301 |
| | $\delta$=1_regOFF | 79 | 252 | 87 | 306 | 384 | 791 |
| | P-Value | 4.92e-43 | 8.23e-76 | 1.59e-45 | 3.26e-188 | 1.25e-183 | 7.15e-320 |
| | $\delta$=2 | 166 | 517 | 175 | 479 | 823 | 1623 |
| | P-Value | 3.11e-70 | 7.37e-102 | 5.29e-68 | 1.53e-187 | 6.18e-309 | 0 |
| **Dataset 4** | Reachable | 983 | 14009 | 1030 | 2673 | 4887 | 20862 |
| | $\delta$=0 | 31 | 220 | 49 | 126 | 198 | 474 |
| | P-Value | 4.10e-15 | 1.48e-45 | 1.44e-30 | 2.29e-75 | 7.67e-106 | 8.81e-152 |
| | $\delta$=1 | 40 | 208 | 89 | 137 | 356 | 819 |
| | P-Value | 3.74e-11 | 3.64e-04 | 3.74e-46 | 2.09e-44 | 2.25e-156 | 1.08e-185 |
| | $\delta$=1_regOFF | 65 | 400 | 93 | 229 | 398 | 912 |
| | P-Value | 1.15e-25 | 2.49e-46 | 1.71e-46 | 2.99e-107 | 1.61e-177 | 4.34e-211 |
| | $\delta$=2 | 159 | 1048 | 202 | 511 | 948 | 2261 |
| | P-Value | 4.33e-50 | 3.82e-90 | 1.74e-76 | 8.03e-186 | 0 | 0 |

(a) Number of relations which were present the databases and *Regulus* output, depending on the deviation ("reachable" means the TF and the gene involve pass *Regulus* expression threshold). NS: Non significant (p-value >0.05) and 0: significant but over the bound of R *binom.test*.

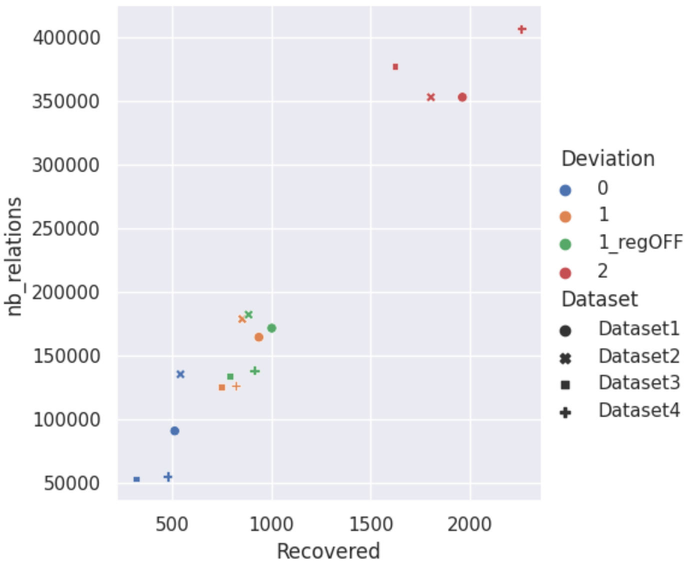


(b) Number of relations found in at least one database compared to the size of the *Regulus* networks while relaxing the likelihood constraint.

**S8 Fig**: **Effect of varying likelihood constraints on the recovery of known regulatory relations.** For each deviation (same as S6 Fig): number of relations which present in the different relations databases (Cytreg, HTRI, Signor, Tfacts or Trrust) or at least in one (ALL). The proportion of the networks recovered is between 0.4 and 0.85% with an average of 0.56%, this number slightly lowers with a relaxed deviation but with no significance (0.6 at $\delta$=0 and 0.51% at 2). Relative to Results subsection *Application to FANTOM5 data*.
