# Supplementary material for: Regulus infers signed regulatory relations from few samples’ information using discretization and likelihood constraints
Source: PLoS Comput Biol. 2024 Jan 22;20(1):e1011816. doi: 10.1371/journal.pcbi.1011816 (PMC10833539; doi:10.1371/journal.pcbi.1011816)
Supplement: S9 Fig — Coherence of signs for the relations inferred by Regulus found in the Trrust and Signor databases. Out of the relations found in Trrust 42 to 63% are either unsigned or signed both ways in different contexts. Relaxing the likelihood constraints slightly reduces the quality of relation signs prediction by Regulus. Relative to Results subsection Application to FANTOM5 data. (PDF) [file pcbi.1011816.s009.pdf]

Relations TF-gene in	Deviation Region cstr ON	Trrust				Signor		Trrust ⋂ Signor		
		Total	True	Unknown	Signed & True	Total	True	Total	Different	True
**Dataset 1**	δ=0	217	39%	50%	78%	43	77%	29	1	69%
	δ=1	432	36%	51%	73%	86	71%	54	2	63%
	δ=1_regOFF	454	37%	50%	75%	93	71%	59	2	66%
	δ=2	867	36%	51%	73%	179	75%	114	2	74%
**Dataset 2**	δ=0	305	37%	52%	78%	50	80%	37	2	73%
	δ=1	408	38%	50%	76%	74	78%	52	2	75%
	δ=1_regOFF	415	38%	49%	75%	75	79%	52	2	75%
	δ=2	730	36%	50%	71%	143	76%	99	2	73%
**Dataset 3**	δ=0	158	37%	51%	75%	42	81%	31	0	81%
	δ=1	369	34%	50%	67%	81	74%	54	0	74%
	δ=1_regOFF	384	34%	50%	68%	87	74%	55	0	75%
	δ=2	823	30%	51%	61%	175	67%	113	1	65%
**Dataset 4**	δ=0	198	42%	45%	76%	49	73%	28	0	79%
	δ=1	356	41%	44%	73%	89	75%	53	1	74%
	δ=1_regOFF	398	38%	46%	71%	93	73%	54	1	72%
	δ=2	948	34%	47%	64%	202	68%	130	4	65%

(a) Relations found in Trrust and Signor and coherence of signs. Total: number of relation in the database and in the network, True: percentage of relations found that have the same sign as the database(s), Unknown: unsigned or differently signed relations or in one database, Signed & True: percentage of relations that are uniquely signed in Trrust also found with the same sign, Different: relations signed differently between the two databases.

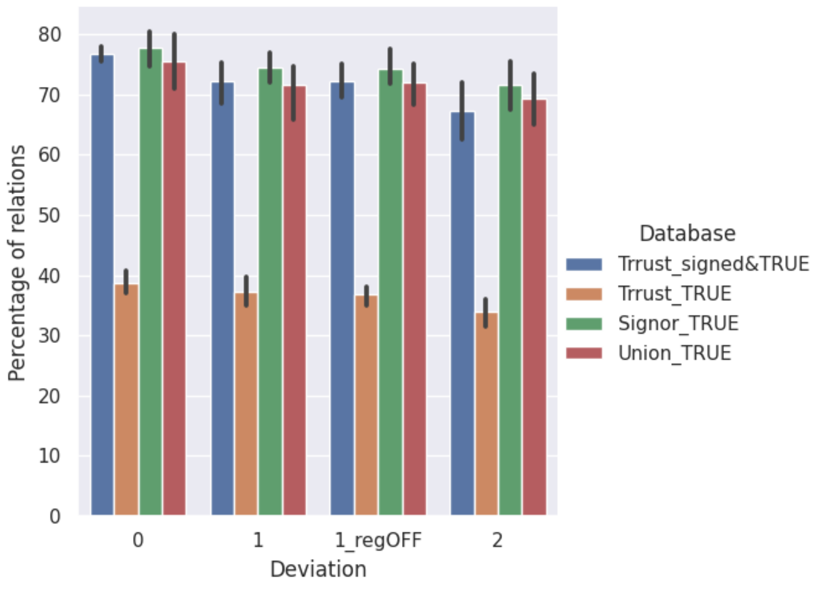

(b) Percentage of relations found having the same sign in Trrust or/and Signor and in the *Regulus* network.Based on the number of Table **S9 Fig**a

S9 Fig: **Effect of likelihood constraints deviation (same as S6 Fig) on the recovery of known directed regulatory relations (activation or inhibition).** Coherence of signs for the relations inferred by *Regulus* found in the Trrust and Signor databases. Out of the relations found in Trrust 42 to 63% are either unsigned or signed both ways in different contexts. Relaxing the likelihood constraints slightly reduces the quality of relation signs prediction by *Regulus*. Relative to Results subsection *Application to FANTOM5 data*.
