# Supplementary material for: Regulus infers signed regulatory relations from few samples’ information using discretization and likelihood constraints
Source: PLoS Comput Biol. 2024 Jan 22;20(1):e1011816. doi: 10.1371/journal.pcbi.1011816 (PMC10833539; doi:10.1371/journal.pcbi.1011816)
Supplement: S10 Fig — (a-c) Distribution of the number of TFs potentially regulating a gene for (a) Regulatory Circuits circuits, uniquely for the reduced datasets used in Fig 2, (b) Regulus circuits before applying the global consistency rules, (c) Regulus final circuits. All data presented here include non-expressed genes, explaining the high value for x = 0. (e-f) Percentage of genes from Roadmap Epigenomics RNA-seq datasets related to the cell populations found in circuits inferred by Regulatory Circuits (e) and Regulus (f) according to their expression level. The datasets are the ones presented in Results subsection Application to FANTOM5 data and Fig 2F. Relative to Fig 3 and to Results subsection Comparison between Regulus and Regulatory Circuits. (PDF) [file pcbi.1011816.s010.pdf]

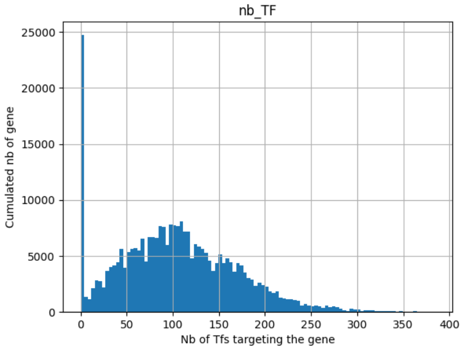

(a)

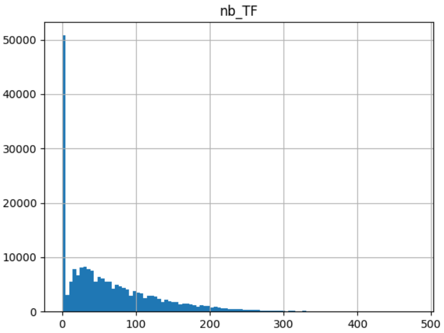

(b)

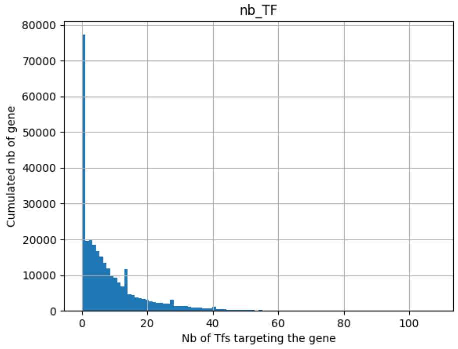

(c)

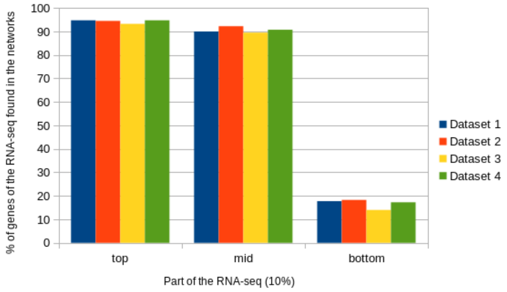

(d)

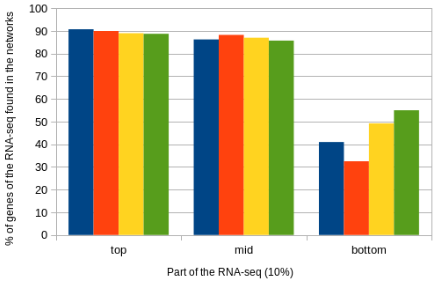

(e)

S10 Fig: **Comparison of circuits between *Regulatory Circuits* and *Regulus*** (a-c) Distribution of the number of TFs potentially regulating a gene for (a) *Regulatory Circuits* circuits, uniquely for the reduced datasets used in Fig 2, (b) *Regulus* circuits before applying the global consistency rules, (c) *Regulus* final circuits. All data presented here include non-expressed genes, explaining the high value for x = 0. (e-f) Percentage of genes from *Roadmap Epigenomics* RNA-seq datasets related to the cell populations found in circuits inferred by *Regulatory Circuits* (e) and *Regulus* (f) according to their expression level. The datasets are the ones presented in Results subsection *Application to FANTOM5 data* and Fig 2F. Relative to Fig 3 and to Results subsection *Comparison between Regulus and Regulatory Circuits*.
