# Supplementary material for: Regulus infers signed regulatory relations from few samples’ information using discretization and likelihood constraints
Source: PLoS Comput Biol. 2024 Jan 22;20(1):e1011816. doi: 10.1371/journal.pcbi.1011816 (PMC10833539; doi:10.1371/journal.pcbi.1011816)
Supplement: S1 Table — a = activity, a(t) time series of the activity, * optional, BS = TF binding site, None = description of the algorithm without implementation. Most methods (11/15) use time series of gene expressions as the only input data, and therefore do not take the regulatory regions activity into account. Few use information about TFs binding sites or regulatory regions (among them, we identified Regulatory Circuits [10]) and only one([34]) checks whether the candidate TFs are expressed. The resulting circuits may then contain relations which are not consistent with the biological situation. Most methods also produce circuits with weighted edges, based on statistical or probabilistic analyses requiring large datasets acquired at several time points, which is a strong limitation to their application to human data. Indeed, many of these methods have only been tested on Escherichia Coli expression data and are limited to small subset of genes, raising the question of their scalability and application to human settings. Finally, we noticed that only the two most recent methods [35, 36] predict the activator or inhibitor role of the inferred regulations to generate signed circuits, but they ignore both TFs expression levels and binding site accessibility. The closest method to what we aim for is Regulatory Circuits, but it still shows some design and reproducibility issues, as shown in the main text. Relative to the Introduction. (PDF) [file pcbi.1011816.s011.pdf]

Method name	Ref.	Data					Data Normalization			Graph		Implementation	Comment
		Genes	Regions	TFs	Other	Nb of samples	2 level	multi-level	Cont.	Scored	Signed		
**REVEAL**	[9]	a(t)					x					None	
**BANJO**	[4]	a(t)				52		x				None	
**NIR**	[3]	a(t)				10*8		x				None	Restricted network (9 genes)
**ARACNE**	[12] [2]	a(t)				336	x			x		None	B cell network
**TSNI**	[1]	a(t)				6*3		x		x		None	Focus on 1 gene
**COALESCE**	[6]	a(t)		BS*	nucleosome positioning*, evolutionary conservation*	125		x		x		C++ implementation & web interface	
**DISTILLER**	[8]	a		BS		870			x	x		Integration: self mining	co-expressed genes
**Mix-CLR**	[10]	a(t)						x		x		None	
**TIGRESS**	[5]	a(t)				907		x		x		Matlab implementation	
**iRafNet**	[15]	a(t)*, a*, Knock-down*		BS*	interaction protein-protein*	17 + 169		x		x		R implementation	
**Regulatory Circuits**	[11]	a	a	BS		808		x		x		Workflow	
**SINCERITIES**	[14]	a(t)				8*100			x	x	x	None	Single cell GRN inference
**Inferelator v3**	[13]	a, a(t)	a	BS	Priors*	254			x	x	x	Fortran / Matlab	
**PoLoBag**	[16]	a(t)							x	x	x	None	
**TRIPOD**	[7]	a	a	motif		80			x			R package	Single cell GRN inference

S1 Table: **Review of current circuit inference methods.** a = activity, a(t) time series of the activity, * optional, BS = TF binding site, None = description of the algorithm without implementation. Most methods (11/15) use time series of gene expressions as the only input data, and therefore do not take the regulatory regions activity into account. Few use information about TFs binding sites or regulatory regions (among them, we identified *Regulatory Circuits* [11]) and only one([7]) checks whether the candidate TFs are expressed. The resulting circuits may then contain relations which are not consistent with the biological situation. Most methods also produce circuits with weighted edges, based on statistical or probabilistic analyses requiring large datasets acquired at several time points, which is a strong limitation to their application to human data. Indeed, many of these methods have only been tested on *Escherichia Coli* expression data and are limited to small subset of genes, raising the question of their scalability and application to human settings. Finally, we noticed that only the two most recent methods [14, 16] predict the activator or inhibitor role of the inferred regulations to generate signed circuits, but they ignore both TFs expression levels and binding site accessibility. The closest method to what we aim for is *Regulatory Circuits*, but it still shows some design and reproducibility issues, as shown in the main text. Relative to the Introduction.

S1 Table References

[1] Mukesh Bansal, Giusy Della Gatta, and Diego Di Bernardo. “Inference of gene regulatory networks and compound mode of action from time course gene expression profiles”. In: Bioinformatics 22.7 (2006), pp. 815–822.

[2] K. Basso et al. “Reverse engineering of regulatory networks in human B cells”. In: Nat Genet 37.4 (2005), pp. 382–390.

[3] Timothy S Gardner et al. “Inferring genetic networks and identifying compound mode of action via expression profiling”. In: Science 301.5629 (2003), pp. 102–105.

[4] Alexander J Hartemink et al. “Using graphical models and genomic expression data to statistically validate models of genetic regulatory networks”. In: Biocomputing 2001. World Scientific, 2000, pp. 422–433.

[5] Anne-Claire Haury et al. “TIGRESS: trustful inference of gene regulation using stability selection”. In: BMC systems biology 6.1 (2012), p. 145.

[6] Curtis Huttenhower et al. “Detailing regulatory networks through large scale data integration”. In: Bioinformatics 25.24 (2009), pp. 3267–3274.

[7] Y. Jiang et al. “Nonparametric single-cell multiomic characterization of trio relationships between transcription factors, target genes, and cis-regulatory regions”. In: Cell Systems 13.9 (Sept. 2022), pp. 737–751.

[8] Karen Lemmens et al. “DISTILLER: a data integration framework to reveal condition dependency of complex regulons in Escherichia coli”. In: Genome biology 10.3 (2009), R27.

[9] Shoudan Liang, Stefanie Fuhrman, Roland Somogyi, et al. “Reveal, a general reverse engineering algorithm for inference of genetic network architectures”. In: Pacific symposium on biocomputing. Vol. 3. 1998, pp. 18–29.

[10] Aviv Madar et al. “DREAM3: network inference using dynamic context likelihood of relatedness and the inferelator”. In: PloS one 5.3 (2010), e9803.

[11] Daniel Marbach et al. “Tissue-specific regulatory circuits reveal variable modular perturbations across complex diseases”. In: Nature methods 13.4 (2016), p. 366.

[12] Adam A Margolin et al. “ARACNE: an algorithm for the reconstruction of gene regulatory networks in a mammalian cellular context”. In: BMC bioinformatics. Vol. 7. Springer. 2006, S7.

[13] E. R. Miraldi et al. “Leveraging chromatin accessibility for transcriptional regulatory network inference in T Helper 17 Cells”. In: Genome Res 29.3 (Mar. 2019), pp. 449–463.

[14] Nan Papili Gao et al. “SINCERITIES: inferring gene regulatory networks from time-stamped single cell transcriptional expression profiles”. In: Bioinformatics 34.2 (2018), pp. 258–266.

[15] Francesca Petralia et al. “Integrative random forest for gene regulatory network inference”. In: Bioinformatics 31.12 (2015), pp. i197–i205.

[16] Gourab Ghosh Roy et al. “PoLoBag: Polynomial Lasso Bagging for signed gene regulatory network inference from expression data”. In: Bioinformatics (2020).
