# Supplementary material for: Regulus infers signed regulatory relations from few samples’ information using discretization and likelihood constraints
Source: PLoS Comput Biol. 2024 Jan 22;20(1):e1011816. doi: 10.1371/journal.pcbi.1011816 (PMC10833539; doi:10.1371/journal.pcbi.1011816)
Supplement: S2 Table — Number of relations present in the different relations databases (Cytreg, HTRI, Signor, Tfacts or Trrust) present in the computed networks. Overall, Regulatory Circuits is enriched in relations found in databases, with significant enrichment obtained in 19 out of 20 cases (p-values ranging from 4.6e-3 to 1.7e-290). Relative to Fig 3. (PDF) [file pcbi.1011816.s012.pdf]

**Dataset**		Cytreg	HTRI	Signor	Tfacts	Trrust
	**Reachable**	1308	14656	1273	3046	5513
**Dataset 1**	Got	143	1744	205	646	1077
815,084	P-values	1.32E-05	8.74E-72	1.92E-22	1.57E-112	6.30E-162
**Dataset 2**	Got	122	1091	187	583	969
448,203	P-values	1.40E-15	9.70E-71	7.84E-47	2.50E-192	1.70E-290
**Dataset 3**	Got	34	622	89	327	536
236,416	P-values	**NS** (> 0,17)	4.75E-51	2.00E-20	1.02E-115	3.20E-170
**Dataset 4**	Got	50	684	97	358	595
278,165	P-values	4.60E-03	1.83E-46	6.62E-20	1.24E-117	3.96E-177

S2 Table: **Recovery of known regulatory relations from *Regulatory Circuits* networks.** Number of relations present in the different relations databases (Cytreg, HTRI, Signor, Tfacts or Trrust) present in the computed networks. Overall, *Regulatory Circuits* is enriched in relations found in databases, with significant enrichment obtained in 19 out of 20 cases (p-values ranging from 4.6e-3 to 1.7e-290). Relative to Fig 3.
