# Supplementary material for: Regulus infers signed regulatory relations from few samples’ information using discretization and likelihood constraints
Source: PLoS Comput Biol. 2024 Jan 22;20(1):e1011816. doi: 10.1371/journal.pcbi.1011816 (PMC10833539; doi:10.1371/journal.pcbi.1011816)
Supplement: S3 Table — Data were processed with a restrictive set of TF binding sites, filtered to have a strictly positive score as found in the supplementary data of [10]. ND: not determined, as all consistent relations involving constantly expressed genes also involve TFs and regions with constant activities; it is therefore not possible to qualify these relations as activation or inhibition. *: percentage taking into account the undetermined relations of the 5555 pattern. Relative to Fig 4. (PDF) [file pcbi.1011816.s013.pdf]

Pattern or Nb of patterns	Nb of genes in the pattern(s)	Percentage among all genes	Nb of TFs in the pattern(s)	Nb of TF-genes relations targeting the pattern(s)	Percentage of all relations	Percentage of activations	Nb of TFs targeting the pattern	Nb of TFs passing the coverage / specificity filter	Percentage of TFs passing the coverage / specificity filter	Biological interpretation
All: 109	26,734	100 %	602	313,627	100 %	50.82 %*	275	121	44.00 %	
Pattern *0000*	14,921	55.81 %	327	0	0 %	0 %	0	0	0 %	Not expressed
Pattern *5555*	3,591	13.43 %	56	56,087	17.88 %	ND %	42	0	0 %	No variations
Pattern *4441*	1,418	5.30 %	63	71,120	22.68 %	64.62 %	186	0	0 %	Decreasing expression in PB
Pattern *1114*	1,244	4.65 %	11	62,082	19.79 %	38.22 %	184	0	0 %	Increasing expression in PB
Patters with more than 100 genes, n = 16	[102-557]<br>mean: 232<br>median: 173<br>sum: 3,704	[0.38-2.08]%<br>mean: 0.87%<br>median: 0.65<br>sum: 13.86%	[0-15]<br>mean: 5<br>median: 3.5	[650–21,026]<br>mean: 6,798<br>median: 3,172<br>sum: 108,764	[0.21-6.70]%<br>mean: 2.17%<br>median: 1.01%<br>sum: 34.68%	[15.78-82.40]%<br>mean: 48.25%<br>median: 44.00%	[54-148]<br>mean: 117<br>median: 121	[10-23]<br>mean: 16<br>median: 15.5	[8.11-20.45]%<br>mean: 14.65%<br>median: 15.66%	Potentially interesting: nb genes
Patters with less than 100 genes, n = 92	[[1-76]<br>mean: 21<br>median: 13<br>sum: 1,856	[0-0.28]%<br>mean: 0.08%<br>median: 0.05%<br>sum: 6.94%	[0-4]<br>mean: 0.74<br>median: 0	[0–1,307]<br>mean: 175<br>median: 56<br>sum: 15,574	[0-0.42]%<br>mean: 0.06%<br>median: 0.02%<br>sum: 4.97%	[0-100]%<br>mean: 53.29%<br>median: 55.56%	[0-111]<br>mean: 35<br>median: 25	[0-17]<br>mean: 5<br>median: 2	[0-33.3]%<br>mean: 10.39% %<br>median: 11.71	Difficult to interpret: low nb of genes

S3 Table: Descriptive statistics on gene expression patterns and TF-gene relations obtained by applying *Regulus* to human B cell subsets. Data were processed with a restrictive set of TF binding sites, filtered to have a strictly positive score as found in the supplementary data of [11]. ND: not determined, as all consistent relations involving constantly expressed genes also involve TFs and regions with constant activities; it is therefore not possible to qualify these relations as activation or inhibition. *: percentage taking into account the undetermined relations of the *5555* pattern. Relative to Fig 4.
