# Supplementary material for: Regulus infers signed regulatory relations from few samples’ information using discretization and likelihood constraints
Source: PLoS Comput Biol. 2024 Jan 22;20(1):e1011816. doi: 10.1371/journal.pcbi.1011816 (PMC10833539; doi:10.1371/journal.pcbi.1011816)
Supplement: Attachment — Submitted filename: ResponseToReviewers.pdf [file pcbi.1011816.s015.pdf]

Response to reviewers' comments (following the comments, in italic font) - PCOMPBIOL-D-22-01850

References to modified or added text are given as line numbers for the final version of the manuscript.

Reviewer #1: The manuscript presents a method ("Regulus") to find regulators of genes and classify these as activators of inhibitors. Using datasets as inputs. Authors claim the method is suitable for experiments with "few" samples, thus filling a gap within the literature.

The problem tackled sounds important, and the solution suggested seems to be elegant, but I cannot see the results presented in an understandable way. I would encourage authors to improve the manuscript. May the following comments be useful:

1) Figures have very low quality. I cannot see but just a portion of the info that is in there. Thus I am not able to judge the results properly.

> We apologize for the inconvenience. All figures have been resized, modified and their quality and resolution were increased. We hope that these improvements will provide an easier reading of the revised manuscript.

2) The solution is not benchmarked against existing tools (or itself). I find table S1 insufficient. Authors claim the other methods are not "easily implemented", which needs more information. How does the tool perform using the same problem and same measurements compared against others? If using "few samples" is the advantage… how many is "few"? Is there a limit? Which one?

> We removed the claim about implementation of other methods and rewrote the introduction to be clearer. It is difficult to benchmark against other methods, as these latter are not adapted to a small number of samples and/or do not take the region accessibility or TF binding into account. In the last subsection of the "Results" section, we compare Regulus to the only applicable method we were able to use and find that Regulus compares favorably, in particular as it provides a sign for the relations (activation or inhibition) and incorporates more low expressed genes (it should be noted that TF encoding genes are often expressed a low levels).

In response to the following comment, we formalized the biological constraints used to filter the relations based on their likelihood (see the response to comment #3 below). In this process, we tested several relaxation values when filtering the relations to measure the consequences on the overall size of the network and the quality of the relations (added paragraphs lines 219-232 and 262-270, added Fig S6 to S9).

As stated by the reviewer, our main goal with Regulus is to provide a method for situations where "few samples" are available. There is no given limit as this was not tested, but the range (10-20 samples representing 4 to 8 cell populations) is now mentioned in Introduction (lines 22-26).

3) Thorough the manuscript authors say to be using “logical consistency check translated from biological knowledge”, “a score which was estimated according to experts” and similar statements to refer to key areas of the method. What do authors mean? Can authors provide a more specific explanation?

> *We thank the reviewer for this insightful comment. To answer it, we changed our working hypothesis (Introduction lines 54-70) and extensively modified the article in the description of the Regulus tool (lines 152-177) and in the Methods section to translate the biological principles into equations (lines 615-651). The two main biological principles are the following:*

- *the maximum effect on gene expression is obtained when the TF is at its highest expression level*
- *The more accessible the region, the higher is the impact of the TF on the gene expression level*

As a basic start point, we consider the situation where the region is the most accessible and therefore allows for regulation. For an activation we have a “perfect” situation if the TF and gene expression levels are the same (dark blue diagonal in Fig. S5a). Then reducing the region accessibility requires more TF expression to get the same target gene expression level (displacement of the dark blue cells in Fig. S5c, e,g). Some flexibility is introduced to take into account the variability of gene expression in response to changes in the TF expression level (light blue and grey cells in Fig. S5) and to modifications of region accessibility.

We hope that this formalization will make our statements easier to understand.

4) “By testing Regulus on unpublished biological datasets”. Why using unpublished datasets? How can readers evaluate performance?

> *We believe this is a confusion based on a mistake in the summary (that has been changed), all the datasets used in this study are published. The datasets are published on GEO since Nov 8th 2020 (submitted Sep 06th 2019): https://www.ncbi.nlm.nih.gov/geo/query/acc.cgi?acc=GSE136988 and https://www.ncbi.nlm.nih.gov/geo/query/acc.cgi?acc=GSE190458.*

References are provided in the B-cells datasets subsection of the “Methods” section (line 728).

5) The species is not mentioned till well into the manuscript. I suggest mentioning what is the species (what cells) being used up front. Also, the fact that the ambition is to target “patho-physiological” conditions and “B cells” should be stated up front. All these are important pieces of info to frame the problem at stake.

> *The species is human, although the methodology proposed by Regulus can be used with other sufficiently well annotated species like mouse, rat, zebrafish or drosophila, as long as users can provide their own TF binding sites coordinates. The species is now mentioned in the summaries and early in the Introduction (line 28), as well as the aim to be used in deciphering patho-physiological conditions (lines 17-26). B cells as a biological use case is also mentioned earlier in the Introduction (lines 27-53).*

6) I would suggest differentiating between the implementation of the tool (Semantic Web, pipeline details, etc.) and the results / performance of the tool. So far all this info is mixed and the resulting manuscript is difficult to read.

> *We made a clearer separation between the Results and the Methods parts although it seemed important to us to have a first Result section describing the main hypotheses at the basis of the Regulus tool and its general functioning scheme.*
As we formalized both the pattern computation and the biological likelihood constraints (see answer to comment #3 above), we also now have a better differentiation between the general principles exposed in the Results section and the implementation of these principles detailed in the Methods section.
As an answer to reviewer #2 major comment #7 we also provide a new Supplementary Figure S1 (S1 Fig) illustrating how the Regulus pipeline works using a "toy example".
Python scripts used to run the pipeline and to implement it are now named in each step of the Methods section.

7) If "entity pattern" is something important (which looks like), then highlight it earlier on. If it is not, I would suggest moving this info to Methods. It is not clear what a pattern is.
> *Entity patterns are now defined early in the Introduction. Their use to apply biological constraints to filter the more relevant TF-gene relations is also presented in the outline of the methodology in Introduction and Results sections. How to obtain the different levels of the patterns is now formalized as equations and more thoroughly described in the Methods section (lines 563-590).*

8) When talking about "computing the distances between them". What do authors mean? Base-pair distances I guess?
> *We do use the base pair distance, the manuscript has been modified to reflect this systematically (lines 119 & 552).*

9) Authors state they are using "four" cell populations from the FANTOM5 dataset. Which ones? Are these NBC, IgM+…? The names of these is a couple pages below. Also, why these ones? How do other tools perform on these specific ones? It looks like table S1 is on a "B dataset" (where is this?)
> *We use four sets of four populations from the FANTOM5 dataset, those are detailed in Fig. 2A. "Regulus was therefore run on four limited datasets extracted from FANTOM5 and used in [10], each containing four cell populations, chosen to be either of similar (comparable organ) or dissimilar (widely different localization) origin." (section Application to FANTOM5 data)*
Regulatory Circuit (Marbach, 2016) inferred regulatory networks on those data and the comparison is presented in Figure 3 and S10 Fig.
The B cells data (NBC, IgM+...) are from another source and available on GEO. They are used to answer to the "use case" presented in Introduction.
Table S1 shows a review of existing methods, the B cell mention is only applicable to the ARACNE method, which was originally developed on B cell data. This is related to the fact that it corresponds to our use case.
All sections in question have been rephrased and mentions about the datasets origin (FANTOM5, e.g.) were added to help the reader identifying which datasets are used in each Results subsection.

Finally, we grouped all the results using the FANTOM5 data (validation of the Regulus pipeline and comparison of its results with those of the Regulatory Circuits project) together and put the application of Regulus to B cell datasets at the end of the Results section. We find that this order is more logical and will make the reading easier.

10) Lines 378 to 387 outline 4 advantages of “Regulus”. I don’t think these advantages are obvious from the text. For instance, take advantage #1 (“under exploitation of the regulatory context”): how under exploited? Any quantification?

> *We meant under exploitation of the regulatory context by other methods. This is now better explained at the beginning of the Discussion section. While some other methods make no use of the TF binding sites or the region accessibility, Regulus uses both (in addition to TF and gene expressions) to infer the regulatory relations. The main characteristics of Regulus have also been better described (lines 445-453).*

Reviewer #2: In this manuscript, the authors report the design of a transcription regulatory inferencing tool, Regulus, which can unravel TF-gene interactions such as activation or inhibition by taking into consideration certain epigenetic information, even with limited data. The idea of using Semantic Web technologies for integrating gene expression data with epigenetic data is neat, and makes it possible to infer context dependent TF-gene interactions making Regulus a powerful tool. The premise is quite interesting, but there are a few more clarifications that are needed about the method, to further strengthen the manuscript.

Major comments:

1. Line 106: "Our circuit inference method is based on the common assumption that genes sharing a common expression dynamics are regulated by a common set of regulators." According to the inferences drawn by Regulus, are the regulatory interactions (activation/inhibition of the genes by the common set of regulators) also same or different for genes sharing common expression dynamics?

> *This is an interesting point and we thank the reviewer for raising it. Actually, regulations can be quite different inside a common gene expression pattern. The relations we consider as interesting are the ones which involve the same TF(s) and gene sharing the same pattern. This issue is indeed answered by the specificity and coverage filter introduced by ClassFactorY: the TFs which according to us are the most probable regulators of one gene pattern, are the ones which regulate similarly most genes of this pattern (high coverage), while not being involved in the regulation of other gene expression patterns (specificity).*

2. Did the authors encounter an example of regulatory inference derived using Regulus that varied with difference in contexts? Highlighting such an example and if possible the implications of wrongly inferring the interactions could be a good addition to the paper.

> *This is an interesting comment and we thank the reviewer for it, as it adds some value to our results. We looked specifically for relations with opposite signs between two FANTOM5 datasets and found that there*

are quite numerous (18% of the shared relations). We now illustrate this fact in the Result section by indicating the number of shared relations and the proportion of those which have opposite signs and by giving a few examples (lines 251-259).

3. Although Regulus helps to zero-in on the most relevant TFs regulating a gene, it does not appear to infer the dynamics of their combined effect. For instance, it does not provide information whether the TFs follow AND, OR or competitive binding. Is this correct -- this should be discussed.
> *We agree with the reviewer's comment and the statement was added in the Discussion section (lines 523-527). This is one of the perspectives of the current work.*

4. The authors claim that Regulus is able to infer the TF-gene interactions with limited data over similar cell types. However, it requires both RNA-seq and ATAC-seq data, which may not be available in many scenarios. Could the authors comment on this?
> *We meant limited data in terms of number, not in terms of techniques. Methods which use only RNA-seq to infer TF-gene relations are usually considered as with lower confidence and it was shown that adding chromatin context to inference methods gives more reliable results (see Miraldi et al, 2019, Genome Res). Accessibility probing by ATAC-seq only requires 50,000 cells and is a commonly performed experiment in molecular biology laboratories. Moreover, large databases of ATAC-seq data are available (such as the ENCODE project, containing 157 human ATAC-seq datasets) and can be used as a proxy when experiments can not be performed (added in Discussion Section, lines 496-503).*

5. In terms of comparisons, there is only a set of comparisons with one tool, Regulatory Circuits. While it is understood that it is the closest method of circuit inference, I am wondering if some predictions can be compared with other databases/tools/studies. This seems to be a slight weakness in this manuscript. Is there a way to more quantitatively compare the results?
> *As stated above in answer to reviewer #1 second comment, we were only able to use Regulatory Circuits on the tested datasets. Other methods either require a great number of samples or we could not implement them. Moreover, as Regulus is devoted to situations where low numbers of cell populations are compared, it was difficult to apply it to large studies. A more quantitative evaluation of Regulus results is performed in the Results section by using several databases to retrieve the inferred TF-gene relations and to confirm their signs (lines 231-268).*

6. Line 459: "All genomic coordinates are given according to the hg19 human reference genome." Why not hg38, which has become the standard for many years now? This will impact the adoption and longevity of Regulus.
> *The methodology used by Regulus can be applied regardless of the version of the genome reference. To accommodate hg38 users we added a hg38 liftover version of the transcription factor binding sites. The*

other inputs are user-defined and are not dependent on any reference genome. This is now mentioned in the Results (lines 113-115) and Methods sections (lines 541-559).
The reason we used the hg19 reference genome is because we wanted to use the Regulatory Circuit database for TF binding sites, which is only available in hg19.

7. At times, I found the paper a bit difficult to read, requiring a bit of back-and-forth between various sections. While it's mostly unavoidable, I am wondering if a toy example, to go with the workflow diagram, will be possible, and can elucidate the methodology better.
> *As stated above in response to reviewer #1 comment 6, we tried to better separate Results and Methods in the revised version of the manuscript and hope that it'll help the reading and understanding.*
In addition, we followed this nice suggestion and now provide a toy example as Supplementary Figure S1 (S1 Fig) to illustrate how the pipeline runs and to explain the main steps. We hope that this new figure will help the reader to follow the sequence of the methodology.

Minor comments:
1. The resolution of 'RDF data model structure' inset in Fig 1 should be improved.
> *All figures were revised and the quality / resolution was improved for all of them. Moreover the RDF data model structure is supplied as a supplementary figure (S3_Fig) for better viewing.*

2. Line 355: 19/20 cases, p-values - could these be supplied in a Supplementary Table?
>*This has been added in a new Supplementary Table (S2 Table, order has changed due to changes in the Results section).*

3. In the label for Fig 3. the Pie chart is marked as B and Table as C but in the Fig, it's the opposite.
> *This has been corrected (Fig 4 caption between lines 359-360, ib.).*

4. Line 484: The number of bins was chosen as four. While this is justified in the text, I wonder if the effect of number of bins has a clear-cut effect on the predictions/performance. Also, it may be interesting to look at the (extreme) Boolean case, as Boolean networks are routinely used to (even if simplistically) model transcriptional regulatory networks.
> *We did not test other numbers of bins, although this is an open issue in the Discussion (lines 461-468). Using boolean would compromise the method used by Regulus as the patterns levels are used to filter the TF-gene relations, and was therefore not tested.*

5. Line 598: less than symbol is not in math mode and hasn't reproduced correctly.
> *This has been corrected*
