# Supplementary material for: Regulus infers signed regulatory relations from few samples’ information using discretization and likelihood constraints
Source: PLoS Comput Biol. 2024 Jan 22;20(1):e1011816. doi: 10.1371/journal.pcbi.1011816 (PMC10833539; doi:10.1371/journal.pcbi.1011816)
Supplement: Attachment — Submitted filename: ResponseToReviewers.pdf [file pcbi.1011816.s016.pdf]

**Response to reviewers' comments (following the comments, in italic font),** PCOMPBIOL-D-22-01850R1

References to modified or added text are given as line numbers for the final version of the manuscript.

Reviewer #1: I went through the revision, and the rebuttal letter. I consider my concerns addressed, so I recommend publication as is.

Reviewer #2: All my original comments have been satisfactorily addressed.

> *We thank both reviewers for their comments on the original version of our work which allowed us to improve the manuscript and to better explain our methodology. We are grateful that this consequent re-writing was considered satisfying and answering the raised concerns.*

Just one additional point to consider: Could the authors specify what is the main bottleneck when applying Regulus to a dataset that has a larger number of samples? Since Regulus incorporates a lot of contextual information in arriving at its inferences, if the method could scale for large datasets, it could potentially be used to validate the results obtained by other inference methods which are more data-centric and are less biology-informed in their approaches.

> *We added a short comment in the discussion (lines 477-480) about the main restriction when applying* Regulus *to larger datasets. We are mainly concerned by the increase in number and complexities of the entity patterns and to the methodology to devise the likelihood constraints in this case.*
